# Template-Based *de Novo* Design for Type II Kinase Inhibitors and Its Extended Application to Acetylcholinesterase Inhibitors

**DOI:** 10.3390/molecules181113487

**Published:** 2013-10-31

**Authors:** Bo-Han Su, Yi-Syuan Huang, Chia-Yun Chang, Yi-Shu Tu, Yufeng J. Tseng

**Affiliations:** 1 Department of Computer Science and Information Engineering, National Taiwan University, 1 Roosevelt Road Sec. 4, Taipei 10617, Taiwan; E-Mail: suborhang@gmail.com; 2 Graduate Institute of Biomedical Electronics and Bioinformatics, National Taiwan University, 1 Roosevelt Road Sec. 4, Taipei 10617, Taiwan; E-Mails: rabbita1101@gmail.com (Y.-S.H.); yishutu@cmdm.tw (Y.-S.T.); 3 College of Medicine, School of Pharmacy, National Taiwan University, 1 Jen-Ai Road Sec. 1, Taipei 10051, Taiwan; E-Mail: chiayun.chang@gmail.com

**Keywords:** template-based, *de novo* design, group efficiency, type II kinase inhibitors, lead optimization, AChE inhibitors

## Abstract

There is a compelling need to discover type II inhibitors targeting the unique DFG-out inactive kinase conformation since they are likely to possess greater potency and selectivity relative to traditional type I inhibitors. Using a known inhibitor, such as a currently available and approved drug or inhibitor, as a template to design new drugs via computational *de novo* design is helpful when working with known ligand-receptor interactions. This study proposes a new template-based *de novo* design protocol to discover new inhibitors that preserve and also optimize the binding interactions of the type II kinase template. First, sorafenib (Nexavar^®^) and nilotinib (Tasigna^®^), two type II inhibitors with different ligand-receptor interactions, were selected as the template compounds. The five-step protocol can reassemble each drug from a large fragment library. Our procedure demonstrates that the selected template compounds can be successfully reassembled while the key ligand-receptor interactions are preserved. Furthermore, to demonstrate that the algorithm is able to construct more potent compounds, we considered kinase inhibitors and other protein dataset, acetylcholinesterase (AChE) inhibitors. The *de novo* optimization was initiated using a template compound possessing a less than optimal activity from a series of aminoisoquinoline and TAK-285 inhibiting type II kinases, and E2020 derivatives inhibiting AChE respectively. Three compounds with greater potency than the template compound were discovered that were also included in the original congeneric series. This template-based lead optimization protocol with the fragment library can help to design compounds with preferred binding interactions of known inhibitors automatically and further optimize the compounds in the binding pockets.

## 1. Introduction

*De novo* drug design usually approaches the creation of new drug-like compounds using a library of building blocks; the library consists of single atoms, functional groups, and small molecular fragments [1]. Instead of evaluating each individual compound, the *de novo* design methodology normally relies on two global optimization algorithms—evolutionary (genetic algorithms [2,3,4,5]) and Monte Carlo-based [6,7,8] methods—to avoid the exhaustive searches. Genetic algorithms evolve an ensemble of potential solutions based on the mutation and crossover principles derived from the concept of natural selection [9]. The Monte Carlo strategy employs the Metropolis criterion [10] to assess whether the resulting change should be probabilistically accepted or rejected in the search for the optimal solutions.

However, *de novo* design methods have typically encountered three main obstacles that have limited their applicability and acceptance within experimental drug discovery programs [4]. The first barrier relates to the synthetic difficulties often associated with the proposed compounds. The second problem is the lack of an efficient search strategy to identify likely fragments to interact within the “druggable space” (binding site). The third and most detrimental drawback is the proposal of compounds with less drug-like properties and/or lower bioactivities than the current collection of compounds. The approaches to improve the synthesizability of suggested compounds can be partially resolved by pre-defined connection rules when assembling new compounds derived from existing drugs or collected from the known organic reaction libraries [11,12,13]. To efficiently search for likely and diverse compounds, evolutionary algorithms [4,14,15] and computational intelligence methods—such as artificial neural networks [16] and fuzzy logic [17]—have been adopted to automatically select features and increase the performance of the compound search. Lastly, to address the issue of low bioactivity, Boda and Johnson [14] assess a compound’s local structure motifs by their occurrence in existing drugs database thus enhancing the drug-likeness of proposed compounds. The overall dogma asserts that if a compound is composed of or contains structural motifs common in known drugs the new compound should also be drug-like.

With a good strategy to identify fragments to possess drug-like properties can greatly improve the lead optimization process. Group efficiency (GE) is a convenient method to assess the relationship between potency and molecular weight. GE estimates the contribution of an individual fragment toward to overall binding energy and assess whether the replacement of fragments are effective in lead optimization process and increasing the bioactivity [18]. GE is derived from the ligand efficiency (LE) that was first presented by Kuntz *et al*. [19] LE is defined as the binding energy per unit of mass (molecular weight) of a given compound [20]. The potency of a ligand to the target is often a key criterion for lead selection. Very often an increase in the molecular weight of a compound corresponds to an increase in its potency [19,21] while unfavorably changing the compound’s drug-like properties such as solubility, metabolic stability, and oral bioavailability [22,23]. Therefore, the balance between potency and molecular weight is important. Empirically, having GE values greater than or equal to 0.3 contributes positively to the ligand’s potency [24] (druggability). Ligand efficiency is routinely used to evaluate potential drug-like compounds due to its ability to effectively balance a compounds potency (bioactivity) and molecular weight [25,26,27].

In this study, we first initiated our method on designing type II kinase inhibitors. Kinase inhibitors are divided into type I and II inhibitors: type I inhibitors bind to the active form of the kinase binding site near the adenine ring of ATP while type II inhibitors bind to a hydrophobic pocket formed by the rearrangement of the DFG-out activation loop—other than the ATP binding site—as is the case with the inactive form of kinase. Due to high structural similarities in the ATP binding pocket, designing highly selective type I inhibitors is difficult [28,29]. On the contrary, the inactive state of kinases are more structurally diverse. By exploring the dissimilarities among the DFG-out conformations [28] structural information can be gathered to help improve compound selectivity with respect to type I and type II kinase inhibitors. Therefore, protocols to *de novo* design selective type II kinase inhibitors are implemented in this study.

Using a known inhibitor or an approved FDA drug as a template to design new inhibitors as part of the lead optimization is helpful since known ligand-receptor interactions can be used as guidance. This study proposes a new template-based *de novo* design protocol to discover new inhibitors that preserve and also optimize the binding interactions of the known type II kinase template. We implement a novel template-based *de novo* design method that incorporates specific binding modes of proven type II kinase inhibitors to construct compounds from large fragments libraries. To demonstrate this method can work for different binding interactions even among Type II inhibitors, two type II inhibitors, sorafenib (Nexavar^®^) [30] and nilotinib (Tasigna^®^) [31], were selected as the first and second templates for the development and validation of our protocol and were specifically selected due to the publicly available crystal structures. The presented five-step protocol is able to reassemble (reconstruct) sorafenib and nilotinib by selecting molecular fragments from a large fragments library. Our procedure demonstrates the ability to reconstruct the two template compounds on different type II inhibitors while keeping their binding interactions with the target.

Furthermore, to demonstrate that the algorithm and associated protocols are able to generate more potent compounds and use in a general lead optimization process, by starting the *de novo* optimization from a known inhibitor, a compound with a lower activity was selected from a series of aminoisoquinoline derivatives, TAK-285 analogues and E2020 analogues [32,33,34] and used as the third, fourth, and fifth template. The aminoisoquinoline derivatives were designed for inhibiting type II kinases B-Raf. TAK-285 analogues and E2020 analogues were designed for inhibiting Human Epidermal Growth Factor Receptor 2 (HER2) and inhibiting AChE. TAK-285 analogues and E2020 analogues were selected as two demo cases that the protocols can also work with protein targets other than the type II kinase. The protocols resulted in the construction of the compounds that are more potent than the starting compound along with being part of the original aminoisoquinoline derivatives, TAK-285 analogues and E2020 analogues collection [32,33,34]. It should also be noted that the creation of these compounds demonstrates the ability of the synthetic feasibility component of the methodology since these compounds were successfully regenerated from a similar starting point (the template).

## 2. Procedure

**Figure 1:**
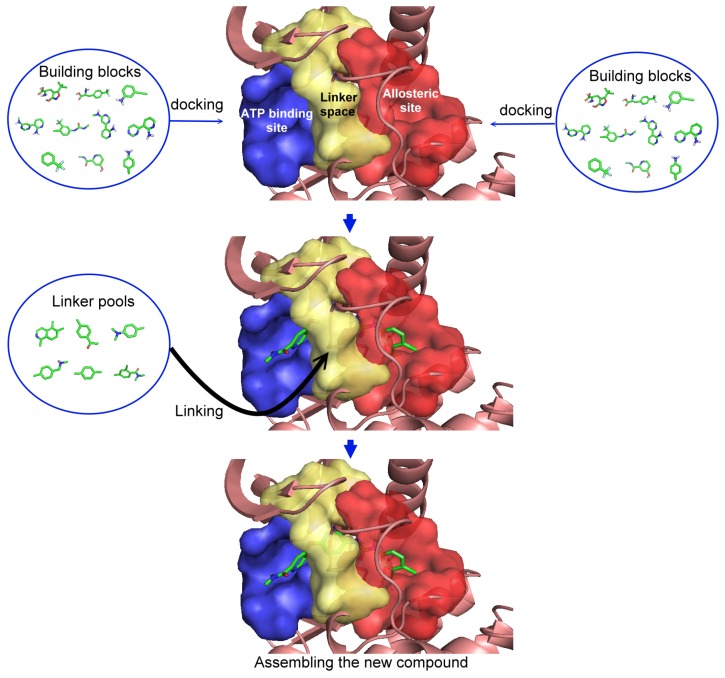
Illustration of the first three steps to generate new compounds based on template molecule.

In this five-step procedure there are three main steps for generation of new compounds (Figure 1) and two last steps for verification of newly constructed compounds. The first step was the selection of a type II inhibitor for the template and the dissection of the binding site according to ligand-receptor interactions. The binding site was dissected into three regions: (i) the allosteric site; (ii) the linker space; and (iii) the ATP binding site. In this study, sorafenib (Nexavar^®^), nilotinib (Tasigna^®^) and a series of aminoisoquinoline inhibitors were chosen as the template compounds. Second, each building block from our in-house fragment library was docked to the ATP binding and allosteric sites, respectively, and evaluated with the group efficiency scores. Third, combining the selected building blocks from the previous steps with all of the possible linkers generated the new collection of compounds. No synthetic accessibility or priorities were considered in this five-step procedure. However, compounds with unstable chemical bonds (synthetically improbable) were removed using a set of molecular filters in the fourth step, and these compounds were then ranked in the last step. These steps resulted in three unique sets of compounds; those based on sorafenib, another set based on nilotinib, and a third group based on a moderately active aminoisoquinoline inhibitor.

### 2.1. Step 1: Indicate the Space (Regions) for Structural Substitution

**Figure 2:**
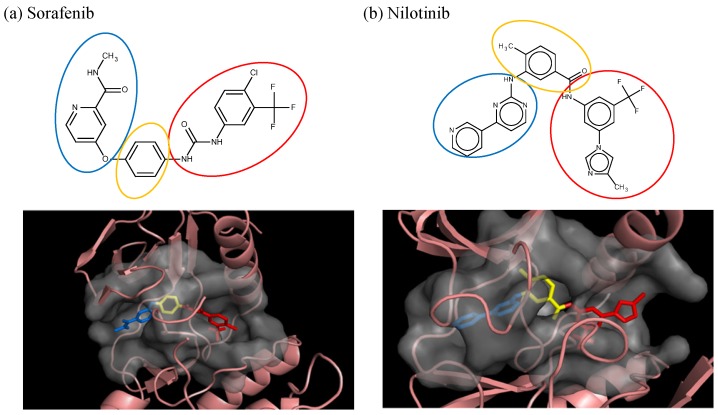
The divided portions of the compounds (**a**) sorafenib and (**b**) nilotinib and their binding structure with B-RAF. The fragment 1 (blue ellipsoid and sticks) in each compound was the ATP site. The fragment 2 (red ellipsoid and sticks) in each compound was the allosteric site. The linker connecting both fragments in each compound was marked as yellow ellipsoid and sticks.

The first step is to divide the binding site into three regions for the tyrosinase kinase examples of this study: the ATP binding site, the linker, and the allosteric site, according to the molecule interactions present in the DFG-out conformation. The ATP binding site is near the hinge region and the allosteric binding site is defined by the DFG-out motif, while the region joining these two binding site features is defined as the linker. Fragments in the ATP binding space are considered the first fragment and fragments in the allosteric space are defined as the second fragment. According to the Liu and Gray’s study [28], the 4-pyridin-3-ylpyrimidin-2-amine of nilotinib was defined as a region binding to the ATP site of receptor (blue in Figure 2(b)) and the 3-(4-methylimidazol-1-yl)-5-(trifluoromethyl)aniline of nilotinib was defined as another region binding to the allosteric site (red in Figure 2(b)). The benzene liking the fragment 1 and fragment 2 of nilotinib was then defined as linker fragment in our study. Because the binding modes of kinase inhibitors are similar, the first fragment of the another example, sorafenib, was defined by a N-methylpyridine-2-carboxamide that binds to the ATP site, and the second fragment of the sorafenib is a 4-4-[4-chloro-3-(trifluoromethyl)phenyl] carbamoylamino that binds to the allosteric site depicted in Figure 1. Figure 2(a) displays each fragments of sorafenib with different colors (blue for fragment 1, yellow for the linker, and red for the fragment 2). The ATP binding region of receptor was then defined by the grid box of 10 Angstroms centered in the geometrical center of the first fragment, and the allosteric region was then defined by the grid box of 10 Angstroms centered in the geometrical center of the second fragment.

### 2.2. Step 2: Evaluate Each Building Block

Each building block is docked into the designated regions for molecular fragment exploration using AutoDock Vina [35] using the default settings. The size of the grid box is set to 10 Å and centered on the geometrical center of the allocated space. The numbers of docked poses is user specified but for the presented examples, the number was set to eight for each building block (molecular fragment). To sufficiently sample the region of interest, fragments are retained based on maximum energy difference between the best binding modes for each fragment. Each fragment undergoes evaluation using group efficiency (GE) scoring. GE was designed to evaluate and probe binding site “hot spots” for increasing affinity and also retained druggability in lead optimization [19,36]. The GE is defined as the change in ∆G (ΔΔG) per non-hydrogen (heavy) atom:

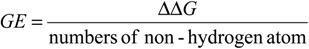
(1)


In this study, the GE scores of each docked fragment were calculated by dividing their respective binding free energy by the numbers of non-hydrogen atoms. In this step, the docked fragment poses having larger or equal (more positive) GE scores and a more negative docking energies or equal than the original fragments of template were selected to construct new compounds. All selected docked fragment poses should fit both conditions of GE scores and docking energies.

### 2.3. Step 3: Assemble to Form New Structures

Linking the docked fragments, from Step 2, to the core (scaffold, building block) of the template with linker fragments assists in the construction of synthetically plausible compounds. Linkers are inserted into the space between the two attachment sites through a series of the geometry transformation. Each linker contains at least two attachment points (denoted as *linkingpoint1* and *linkingpoint2*). All combinations of each two (or more) possible linker attachment points are attempted when performing the geometric transformation to connect linker to the two fragments defined in step 1 (denoted as *fragment1* and *fragment2*). In the first step of the transformation procedure, the linker is translated from *linkingpoint1* to the nearest atom of *fragment1*. The next step is to calculate two vectors originating at *linkingpoint1*—one directed to the atom of *fragment2* which is nearest to *linkingpoint2* (called *vector1*) and the second to *linkingpoint2* (called *vector2*). The linked structure is then rotated from *vector2* to *vector1*. When the distance between the endpoints of *vector1* and *vector2* is acceptable, a new structure is generated from the linker’s rotated combination of the two docked fragments. To reduce the amount of time spent trying to determine the optimal linker orientation, linkers are only included when the distance between attachment points is less than 0.1 Angstroms. The distance limitation can be adjusted to include more molecules. All of these generated structures are then collected as a structure pool for step 4 and step 5.

### 2.4. Step 4: Apply Drug-Likeness Filters

A modified version of Lipinski’s Rule-of-Five [37] is applied to the newly constructed compounds to reduce the number of potential compounds returned to the user for consideration. The modified criteria increase the molecular weight threshold to a maximum of 550 grams per mole (g/mol).

### 2.5. Step 5: Prioritize the New Structures

After generating a collection of new compounds, they are ranked by the summing the GE scores of the replacement fragments calculated in Step 2. A more positive GE score implies better binding affinity to the target whereas lower GE scores results in a new compound being ranked lower for further development.

## 3. Results and Discussion

### 3.1. Sorafenib Reassembly

The sorafenib-B-RAF complex was used to test the algorithm’s ability to rebuild the ligand-receptor molecular interactions of the initial template given a library of fragments. The methodology and protocol of the algorithm would be considered sound if it is able to reconstruct the template molecule—along with other combinations of fragments resulting in other known compounds—since favorable interactions are selected. Each building block was docked into the ATP binding and allosteric site of B-RAF and a total of eight docked poses were selected for each building block to be evaluated. The GE score for each docked fragment was calculated by dividing the docking energy of each pose with the number of non-hydrogen atoms. The docking energy of the fragment 1 (ATP binding site) and the fragment 2 (allosteric binding site) of sorafenib are −5.1 and −6.1 kcal/mol, respectively, with the GE scores being 0.46 and 0.40 for fragments 1 and 2, respectively (Table 1).

**Table 1:** GE scores and docking energies of sorafenib and nilotinib.

Compound	GE score of fragment 1	GE score of fragment 2	Docking energy of fragment 1 (kcal/mol)	Docking energy of fragment 2 (kcal/mol)
**Sorafenib**	0.46	0.40	−5.1	−6.1
**Nilotinib**	0.60	0.39	−7.8	−6.6

The settings for our algorithm considered docked fragments possessing more positive GE scores and (negative) docking energies—than the original parts from the template—to construct new structures. The GE scores for the original sorafenib fragments become the filtering criteria for selecting docked fragments. A total of 5,831 docked fragments from the ATP binding site and 15,055 docked fragments from the allosteric site (out of 27,400 possible poses) were retained. The selected poses of the molecular fragments docked to the ATP binding and allosteric sites along with 38 linkers were used to build the new compounds. Connecting the two docked fragments from each site with linkers to the scaffold, satisfying the required bonding angles and length, generated 101,427 new compounds. The summations of GE scores for the two docked fragments for each new compound were used to rank the compounds. The results demonstrate that sorafenib could be reassembled while retaining the template specified intermolecular interactions, the original sorafenib-B-RAF molecular interactions. Since only fragments possessing better or equal GE and a more negative docking energies or equal than the fragments of sorafenib were used to generate the new compounds, sorafenib is the lowest ranked (least active compound) among the newly generated compounds. Moreover, those newly generated compounds were compounds considered to have better sorafenib-like binding ability.

**Figure 3:**
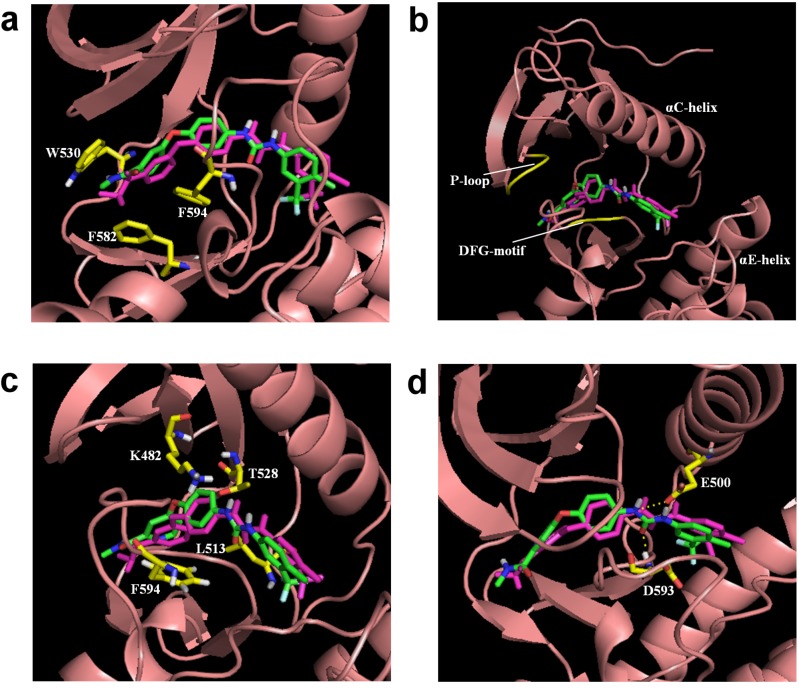
The comparison of regenerated sorafenib to the sorafenib from the crystal structure (in purple) with B-RAF; three hydrophobic interactions within the (a) ATP binding pocket, (b) allosteric site, (c) linker space, and (d) two hydrogen bond interactions.

To determine if the regenerated sorafenib could adopt and retain the same binding mode and interactions as shown in the literature [38], the regenerated sorafenib was docked into the B-RAF crystal structure and subjected to molecular dynamics simulation (MDS). Regenerated sorafenib positioned itself between the ATP binding site and the allosteric site and showed high similarity (RMSD: 1.5 Å) with the crystal structure of sorafenib from PDB ID: 1 uwh (Figure 3 in purple). The bound pose of our regenerated sorafenib and receptor of a snapshot in our MDS taken from the last picosecond interval (1,000th step) was shown in Figure 3. Figure 3(a-c) depict the three hydrophobic interactions of the generated sorafenib-B-RAF complex, the same three key hydrophobic interactions reported in the literature [38]. Figure 3a shows the distal pyridyl ring of sorafenib’s fragment 1 located in/near the ATP binding pocket and interacting with three aromatic residues (W530, F582, and F594). Figure 3(b) illustrates that the lipophilic trifluoromethyl phenyl ring at the end of fragment 2, for the regenerated sorafenib, binds to a hydrophobic cavity formed between the αC and αE helices, the N-terminal regions of the DFG motif and the catalytic loop. Figure 3(c) shows the central phenyl ring of our reconstituted sorafenib contacts three aliphatic side chains K482, L513, and T528 and interacts with F594. In addition to the hydrophobic interactions, the two hydrogen bonds—one between an amide nitrogen and the carboxylate side chain of E500 and the other a carbonyl moiety (of the ligand) to the backbone nitrogen of D59—are also seen from our regenerated sorafenib-B-RAF complex and these computationally observed interactions are consistant with the experimentially derived interaction seen in the crystal complex and literature (Figure 3(d)). From these observation, the algorithm was able to reconstruct the interactions of the original template ligand with the receptor and suggest potentially better inhibitors that possess similar ligand-receptor interactions.

### 3.2. Nilotinib Reassembly

To further probe the abilities of the algorithm, nilotinib a different type II inhibitor and known ABL inhibitor was investigated. Similar to the sorafenib experiment, each building block was docked into the ATP binding and allosteric sites of ABL. A total of eight poses at each site were selected for 3,425 building blocks. As reported in the literature, the 4-pyridin-3-ylpyrimidin-2-amine of silotinib binds to the ATP site (blue in Figure 2(b)) and the 3-(4-methylimidazol-1-yl)-5-(trifluoromethyl)aniline of nilotinib binds to the allosteric site (red in Figure 2(b)). These functional groups (building blocks) were selected as fragment 1 and fragment 2 in this example. The next step was calculating the binding energy and GE scores for each docked fragment. The calculated (predicted) energies of fragments 1 and 2 of nilotinib docked to ABL were −7.8 and −6.6 kcal/mol, respectively, while the GE scores of nilotinib fragments 1 and 2 were 0.60 and 0.39, respectively (Table 1). Of the 27,400 docked fragments, 2,468 fragments at the ATP binding site and 278 fragments at the allosteric site were selected as candidates for the generation of new potential ABL inhibitors. The new compounds were constructed from the two sets of docked fragments using the 38 linker-fragments that satisfied the geometrical constraints; resulting in 2,953 new compounds. The initial template compound of nilotinib was successfully re-generated by linking fragments 1 and 2 with 4-methylbenzaldehyde (a linker). Again, since only fragments having better or equal GE and a more or equal negative docking energies than the fragments of nilotinib were used to generate the new compounds, the reassembled nilotinib is the lowest ranked as in the case of sorafenib. Based on the ability of our algorithm to reconstruct nilotinib in the ABL binding site, it is postulated that this method should be able to aid in the design of kinase specific type II inhibitors. Both the nilotinib and sorafenib examples were used to demonstrate how the method works, none of the other assembled inhibitors in these two examples were tested for their therapeutic effects.

**Figure 4:**
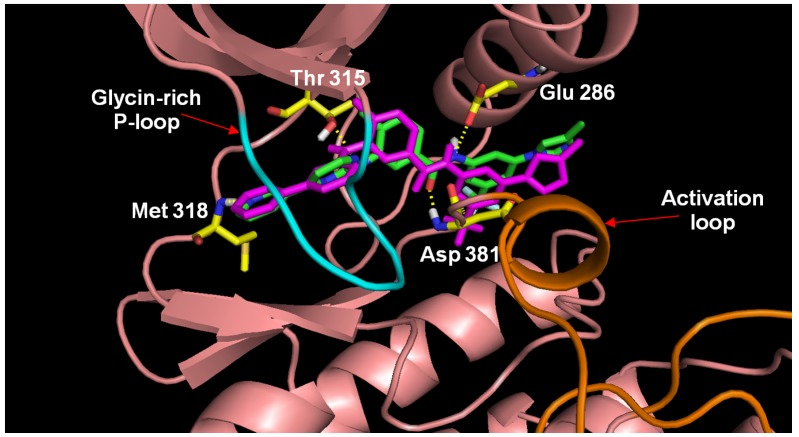
Four hydrogen bonds and one liphophilic bond interactions between regenerated nilotinib and the Abl kinase structure. Fragment 1 of nilotinib is located in a glycine-rich P-loop (cyan color) and fragment 2 of nilotinib is positioned near the activation loop (orange color).

To validate that the reassembled nilotinib retains the same ligand-receptor interactions as those shown in the solved X-ray structure, the regenerated nilotinib was docked to the ABL crystal structure and the best scoring poses was subjected to 1 ns of MDS. The bound pose of our regenerated nilotinib and receptor of a snapshot in our MDS taken from the last picosecond interval (1,000th step) was shown in Figure 4. Figure 4 indicates that fragment 1 interacts with the glycine-rich region of the P-loop fold (cyan sticks; ATP binding site) and fragment 2 approaches the activation loop of ABL (orange sticks; allosteric site). The reconstituted and docked nilotinib exhibits a highly similar bound conformation (RMSD: 1.4 Å) to that of the nilotinib found in the crystal (PDB ID: 3cs9; purple sticks). The four key hydrogen bonding interactions of the nilotinib-ABL complex are retained and include (i) the amino-NH of fragment 2 of and the sidechain carboxylate functional group of Glu286; (ii) the aniline-NH of fragment 1 and the hydroxyl group of Thr315; (iii) the pyridyl-N of fragment 1 interacting with the amide of Met318’s backbone, and (iv) the amido-C=O of fragment 2 and the backbone amide of Asp381. In addition to the hydrogen bonds, an essential lipophilic interaction between the backbone carboxyl group of Asp381 and a fluorine atom in the trifluoromethyl group of the reconstructed nilotinib (illustrated in yellow dotted lines in Figure 4) is preserved. This example demonstrates the successful reconstruction of nilotinib, a type II inhibitor, with our algorithm.

### 3.3. Optimization on the Series of Aminoisoquinoline Derivatives

The sorafenib and nilotinib examples demonstrate the ability to reassemble these different type II kinase inhibitors retaining the required ligand-receptor interactions. A third type II inhibitor was selected from a series of aminoisoquinoline derivatives consisting of ten compounds designed as mutant B-RAF type II inhibitors with IC_50_ values ranging from 1.6 to 110 nM; compound 13 (IC_50_ = 56 nM) was selected as the template compound for this example [32]. Since the building block library includes the fragments contained in this series of compounds, our algorithm should be able to generate compounds that are more potent than Compound **13** in addition to Compound **13**; see Table 2. The left portion (blue ellipsoid in Table 2) of the aminoisoquinoline was selected as fragment 1 and the red ellipsoid denotes fragment 2 (also in Table 2). The docking energies of the original fragments—fragments 1 and 2 of Compound 13 (the template)—were −6.3 and −3.8 kcal/mol, respectively; with GE scores of 0.42 and 0.48. After selecting the docked fragments possessing the greatest positive GE scores and most negative docking energies (compared to the original fragments) for the ATP and allosteric binding sites, 1,429,973 new compounds were constructed. Three (Compounds **1**, **12a** and **15**) of the seven compounds with better IC_50_ values from the original aminoisoquinoline derivatives [32] were identified and are listed in Table 2. The four compounds (out of seven) from the aminoisoquinoline set were not generated in our algorithm since their docked fragment cannot be assembled from the linkers according to our linking criterion described in Section 2.2. The total GE scores (and the ranking within generated compounds) of each fragment for compounds **1**, **12a**, **13**, and **15** were 1.09 (23,490), 1.0 (239,623), 0.95 (620,424), and 1.0 (239,623), respectively. The compounds were ranked by their calculated binding energies and this order directly correlates with their IC_50_. This example demonstrates that our algorithm is able to suggest more potent compounds for the mutant B-RAF kinase when starting with a less potent template compound.

**Table 2:** Ranking of aminoisoquinoline series of compound after optimization. The ellipses define fragments of for each site in Compound **13**; blue for fragment 1 and red for fragment 2.

Rank	B-RAF, IC50 (nM)	Structure	Binding Energy (kCal/mol)	Sum of GE Score	Docking Energy (KCal/mol)
**1**	1.6	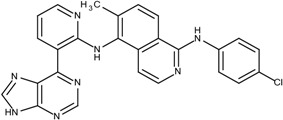 1	−33.0	1.09	−12.4
**2**	17	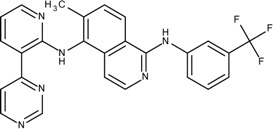 12a	−27.2	1	−11.4
**3**	56	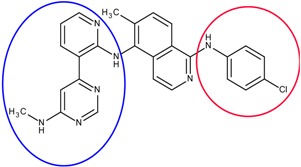 13	−27.0	0.95	−11.5
**4**	18	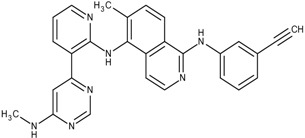 15	−26.7	1	−11.3

### 3.4. Optimization on the Series of TAK-285 Analogues Inhibiting HER2 Kinase

As an additional demonstration of type II kinase inhibitor assembly, TAK-285 was the fourth type-II kinase inhibitor selected for demonstrating our methods in optimization. The TAK-285 series were developed by Ishikawa *et al*. as the HER2/EGFR dual inhibitors [33]. However, we only demonstrated the optimization of HER2 inhibiting activity in order to present the ability of assembling compounds with better activity inhibiting a specific protein target. From Ishikawa’s study, there were 39 compounds developed with pyrrolo[3,2-*d*]pyrimidine scaffolds, with HER2 inhibition IC_50_ values ranging from 2.2 nM to 720 nM [33].

The template compound selected for this demonstration was compound **6n**, which has HER2 inhibition IC_50_ value of 26 nM. The docking scores of the template compound **6n** were −4.4 kcal/mol for fragment 1 and −4.5 kcal/mol for fragment 2, which converted to GE scores of 0.44 for fragment 1 and 0.46 for fragment 2. After the compound assembling and binding energy estimation using MDS, there were seven compounds in the original paper [33] within the assembled compounds which had higher HER2 inhibition activity than compound **6n** (Table 3)

**Table 3:** Ranking of TAK-285 analogues of compound after optimization. The ellipses define fragments of for each site in Compound **6n**; blue for fragment 1 and red for fragment 2.

Rank	HER2, IC50(nM)	Structure	Binding Energy (kCal/mol)
**1**	20	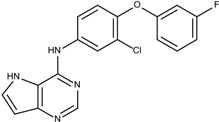 6c	−21.1
**2**	4.1	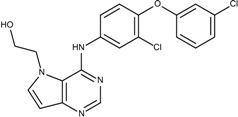 10e	−21.0
**3**	4.6	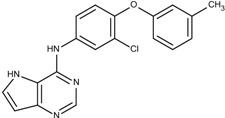 6m	−20.9
**4**	26	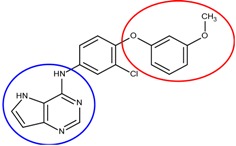 6n	−19.4
**5**	8.3	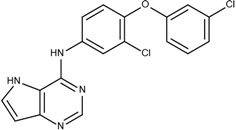 6e	−19.2
**6**	17	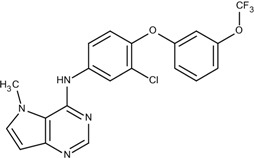 8l	−17.0
**7**	12	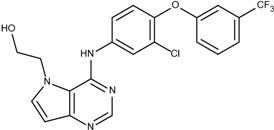 10j	−16.7
**8**	3.3	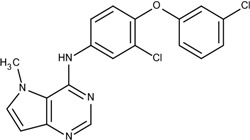 8e	−15.3

**Figure 5:**
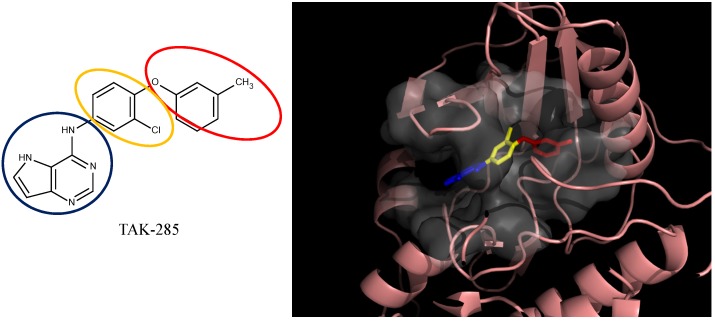
The compound **6m** from the TAK-285 series docked with HER2 kinase (PDB ID: 3RCD). The ellipsoids define the portion of the compounds that interact with the HER2 binding site—ATP site (blue ellipsoid; fragment 1) and allosteric site (red ellipsoid; fragment 2)—while the linker is denoted with the orange ellipsoid. The cavity of HER2 is depicted as a gray surface with a bound inhibitor (tube). The inhibitor is represented in a similar fashion as the depicted compounds. The portions of the compound that interacts with the different regions of the binding site are color-coded. The portion that interacts with the ATP binding site is blue (fragment 1), the linker is yellow, and the allosteric site portion is red (fragment 2).

**Figure 6:**
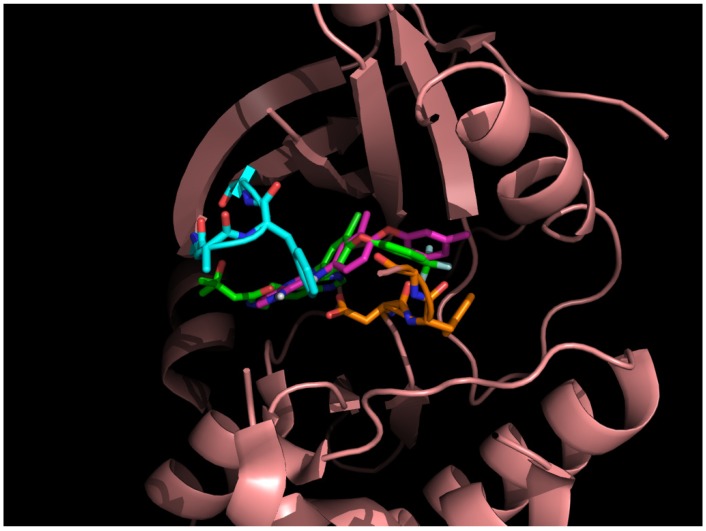
The docked compound **6m** (purple) compared to the TAK-285 (green) within the HER2 crystal structure (PDB ID: 3RCD). The kinase P-loop (cyan) and DFG-motif (orange) were indicated.

In the following docking demonstration, we selected compound **6m** as an example. The docking scores for each fragment of compound **6m** were both −4.4 kcal/mol; but the GE scores were 0.44 and 0.55 respectively because of the difference of the atom counts. For the analysis of the interaction between the compound **6m** and the HER2 kinase, we performed a docking study with the structure of compound **6m** and the HER2 crystal structure (PDB ID: 3RCD). The best docking pose of compound **6m** which has docking score of −9.9 kcal/mol, as shown in Figure 5. Moreover, we also compared the docking pose and the TAK-285 (compound 34e, HER2 IC_50_: 17 nM) structure, which was crystallized with HER2 kinase in the 3RCD (Figure 6). Within the ATP site, the compound **6m** only had the interaction with the phenylalanine of the P-loop because it does not have extending chain on the pyrrole ring, compared to TAK-285. On the other side, within the allosteric site, the fragment 2 of compound **6m** still had hydrophobic interaction with DFG motif.

To sum up in this case, we have demonstrate that the compound **6n**, which is the compound has the lower HER2 inhibiting activity, can be optimized into compounds with better activity using our *de novo* fragment assembling methods. We also demonstrated the potential binding interaction between one of the assembled compounds and the HER2 kinase using the docking analysis.

### 3.5. Optimization on the Series of E2020 Analogues

The acetylcholinesterase (AChE) inhibitor was selected from a series of E2020 analogues consisting of thirteen compounds with IC_50_ values ranging from 0.9 to 4,400 nM; compound **22** (IC_50_ = 4,400 nM) was selected as the template compound for this example [34]. Since the building block library includes the fragments contained in this series of compounds, our algorithm should be able to generate compounds that are more potent than Compound **22** in addition to Compound **22**; see Table 4. The left portion (blue ellipsoid in Table 4) of the dihydronaphthalene was selected as fragment 1 and the red ellipsoid denotes fragment 2 (also in Table 4). The docking energies of the original fragments—fragments 1 and 2 of Compound **22** (the template)—were −5.0 and −4.1 kcal/mol, respectively; with GE scores of 0.69 and 0.59. After selecting the docked fragments possessing the greatest positive GE scores and most negative docking energies (compared to the original fragments) for the binding sites, and about one hundred thousand new compounds were constructed. Three (Compounds **18B**, **25** and **26**) of the twelve compounds with better IC_50_ values than the template, compound **22**, from the E2020 analogues were identified and are listed in Table 4. The eight compounds from the E2020 series set were not generated in our algorithm since their docked fragment cannot be assembled from the linkers according to our linking criteria. The total GE scores of each fragment for compounds **18B**, **22**, **25** and **26** were 1.28, 1.15, 1.2, and 1.17, respectively. The compounds were ranked by their calculated binding energies, but the ranking of the binding energies for the compounds **18B** and **26** were not as expected. However, the experimental IC_50_ values for the two compounds were close. This was an acceptable error.

**Figure 7:**
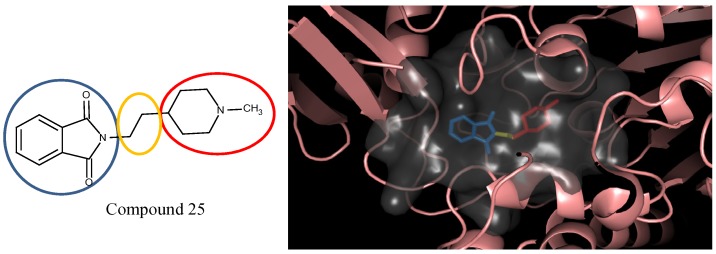
The divided Compound **25** of E2020 series. The ellipsoids define the portion of the compound that interact with the AChE binding site –the peripheral site (blue ellipsoid and sticks; fragment 1) and catalytic site (red ellipsoid and sticks; fragment 2)—while the linker is denoted with the orange ellipsoid. The cavity of AChE is depicted as a gray surface with a bound inhibitor (tube).

**Figure 8:**
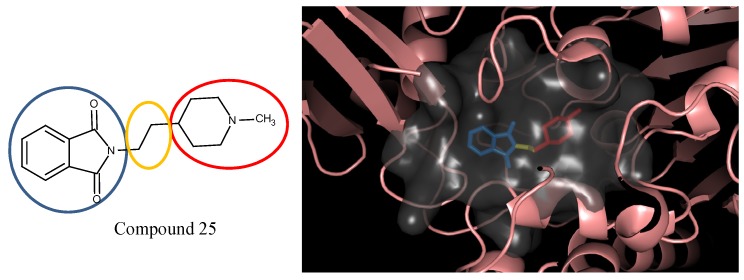
The regenerated compound **25** of E2020 series and bound to AChE crystal structure; three hydrophobic interactions within the (**a**) peripheral pocket, (**b**) catalytic site, (**c**) linker space, and (**d**) hydrogen bond interaction.

In the compound **25**, the isoindole-1,3-dione was the region binding to the peripheral site of receptor (blue in Figure 7) and the 1,4-dimethylpiperidine of compound **25** was the region binding to the catalytic site (red in Figure 7). The ethane liking the fragment 1 and fragment 2 of compound was then defined as linker fragment in our study. To demonstrate the interactions between the binding sites and the regenerated compound **25**, the ligand was bound to the receptor, AChE, crystal structure (PDB ID: 1b41) as shown in Figure 8. Figure 8(a) and Figure 6(b,c) depict the hydrophobic interactions between the two cavities and the linker region and the generated compound **25**-AChE complex. Figure 8(a) shows the fragment 1 of the compound **25** bound to a hydrophobic peripheral cavity and interacting with two aromatic residues (W286 and Y72). Figure 8(b) illustrates that the fragment 2, for the regenerated compound **25**, located in/near the catalytic site and interact with two residues, W86 and F338. Figure 8(c) shows the linker contacts interact with D74. In addition to the hydrophobic interactions, the one hydrogen bond between an aldehyde group in compound 25 and the hydroxyl group of the side chain in Y124 (Figure 8(d)).

**Table 4:** Ranking of E2020 series after optimization. The ellipses define fragments of for each site in Compound **22**; blue for fragment 1 and red for fragment 2.

Rank	AChE, IC50 (nM)	Structure	Binding Energy (kCal/mol)
1	0.9	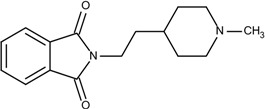 25	−10.23
3	7.7	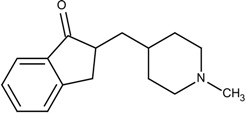 18B	−9.65
2	4.2	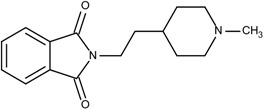 26	−9.47
4	4400	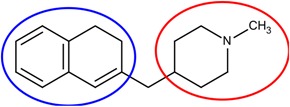 22	−7.73

## 4. Experimental

### 4.1. Datasets

Sorafenib is a potent type II kinase inhibitor that binds to wild type and V599E mutant B-RAF as well as other tyrosine kinases such as VEGFR-2, VEGFR-3, PDGFR, Flt-3, and c-KIT [39,40,41]. The solved X-ray structure of sorafenib complexed to wildtype B-Raf was selected as the first example (PDB ID: 1uwh) [38]. Another potent type II inhibitor, nilotinib, is an aminopyrimidine inhibitor for the Bcr-Abl tyrosine kinase [42], an important kinase for the treatment of chronic myelogenous leukaemia (CML) and Philadelphia chromosome positive (Ph+) acute lymphoblastic leukaemia (ALL) [43,44,45,46] plus it is also a multi-kinase inhibitor for c-Kit and PDGFR [47]. Nilotinib is known to interact with the inactive DFG-out conformation of the *ABL* kinase domain, thus it is also a type II inhibitor [48]. The solved X-ray structure of Bcr-Abl complexed with nilotinib (PDB ID: 3cs9) [31] was used as the starting point for the second example. Using known, potent inhibitors to generate new compounds provides a method to determine if the compound selection protocol is able to adequately select a known inhibitor from a collection of compounds generated from similar building blocks.

The third example is a series of aminoisoquinoline inhibitors that target the V600E B-Raf mutant [32]. More than 70% of melanomas and benign nevi contain a mutation at position 600 (V600E) of B-Raf [49]. This series of aminoisoquinolines are designed as type II inhibitor that binds to the ATP and allosteric sites of the mutant V600E B-RAF. For this example, compound **13** was selected as the template (starting compound) to demonstrate the optimization process (PDB ID: 3idp) [32].

The fourth example is for another type II kinase, Human Epidermal Growth Factor Receptor 2 (HER2), as a demonstration for another type II kinase case. The TAK-285 analogues were designed as HER2/EGFR (Epidermal Growth Factor) dual kinase inhibitors [33]. In this case, we only use the HER2 (PDB ID: 3rcd, 3pp0) as the target for activity optimization. Compound **6n** of the TAK-285 analogues were selected as the template.

The fifth case study is a series of E2020 analogues that inhibit the acetylcholinesterase (AChE) activity [34]. Inhibition of AChE could lead to the accumulation of acetylcholine, a neurotransmitter, and research has found that acetylcholine levels are related to many diseases, including Alzheimer’s disease and Lewy body dementia [50,51]. In this case, compound **22** was selected as the template (starting compound) to demonstrate the optimization process (PDB ID: 1b41) [34,52].

The building block library contains 1,334 structural fragments from the SciFinder database that matched the query “amine” and 2,091 organic building blocks from the commercially available Sigma-Alderich Co. product library (Sigma-Alderich Chemie GmbH, Steinheim, Germany). These building blocks include the typical building blocks in a chemical synthesis such as various nitrogen compounds (amines, isocyanides) and carbonyl compounds (amides, aldehydes, and ketones). Also added to the building block library were the molecular fragments of the selected templates and their analogues since the SciFinder collections did not cover the fragment-space of the template molecules. Additionally, 18 commonly seen linkers were selected from our in-house library and another 20 most common used linkers from Molinspiration [53] were used. Each linker contained, on average, three possible bonding locations (attachment points). Although potential fragment space is infinite, the number of commonly commercial available fragments is limited especially only for lead optimization. In our study, 3,425 different structural diverse fragments and 38 commonly used linkers are selected to demonstrate the lead optimization of type II kinase inhibitors based on the known inhibitors.

### 4.2. Molecular Dynamics Simulation

To determine whether the compounds generated (suggested) by our algorithm exhibit similar intermolecular interaction compared to the selected template compound and refining strucures, 1 nanosecond (ns) molecular dynamics simulation (MDS) were performed for each proposed compound-receptor complex. Because the bonds between the docked fragments and linker of our final generate structures might be in unstable angles, the bound pose of our generated structures were refined by geometry optimized with the MM+ force field in Hyperchem 7.0 [54] using Polak-Ribiere algorithm [55] in advance, and re-docked into their respective receptors in the geometric center of generated complex using AutoDock Vina in default settings. The best complex pose was selected with the favorable contacts between the docked pose of the energy-minimized constructed compounds and the residues within the binding site. The MD simulation was performed using Gromacs 4.03 [56] with the Gromos 53a6 force field. The molecular system was placed in a cubic box of simple point charge (SPC) [57] explicit water molecules and the distance between protein and each edge of the box was set as 0.9 nm. To maintain overall electrostatic neutrality and isotonic conditions, Na^+^ and Cl^−^ ions were randomly positioned within this solvation box. The system was energy minimized using 1,000 iterations of the steepest descent protocol with an energy minimization convergence criteria of a between-step difference smaller than 1,000 kJ mol^−1^ nm^−1^. After the energy minimization, the system was subjected to a 1,000 ps molecular dynamics simulation at constant temperature (300 K), pressure (1 atm), and a time step of 0.002 ps (2 fs) with the coordinates of the systems recorded every 500 steps. To estimate the ligand binding energies, the energy records of last 100 ps were extracted and binding energies were calculated with linear interaction energy method [58].

## 5. Conclusions

In summary, we developed a template-based *de novo* design strategy to create novel type II inhibitors based on the known type II kinase inhibitors such as sorafenib (Nexavar^®^) and nilotinib (Tasigna^®^). Type II kinase inhibitors not only bind to the ATP site but also strongly interact with an allosteric binding site that only occurs in the inactive form of the enzyme. In this study, we defined the ATP and allosteric binding sites along with the linker space, the region between these two sites, as the three key interaction groups. In this method, the five-step protocol was designed to first define the key interaction regions, then dock a library of building blocks (molecular fragments) into each interaction region, assemble the new compounds by linking each potential fragments with the retained core of the initial compound, retain compounds based on drug-likeness properties, and finally prioritize compounds using the calculated GE scores. To reduce calculation time and combinatorial-explosion, the GE scores were employed to rank the new compounds that were then filtered by Lipinski’s Rule-of-Five for drug-likeness. Our algorithm and the discussed protocol successfully rebuilt two *different* type II inhibitors—sorafenib and nilotinib—using an automated and systematic protocol.

In our template based method, the influence of the original complex structure is embedded. First the compound-protein complex itself would affect the docking positions for the fragments and linkers and further affect the GE scores that determine the ranks of the final compounds. Since this method is a “template” based method, it does not work with apo structure although one could eventually modify the method to work with apo structure.

In order to validate that our *de novo* design methods can be applied in other type II kinase cases, a series of aminoisoquinoline derived type II mutant B-RAF inhibitors with IC_50_ values varying from 1.6 to 110 nM, and TAK-285 analogues inhibiting HER2 kinase, with IC_50_ values from 2.2 nM to 720 nM were selected to demonstrate how the our algorithm can discover compounds that are more potent than the provided template compound while possessing similar ligand-receptor interactions [32,33]. An aminoisoquinoline-based compound with an IC_50_ value of 56 nM and a pyrrolo[3,2-d]pyrimidine compound with an IC_50_ value of 26 nM were used as the templates, respectively. The algorithm identified these compounds from the original datasets [32,59] with more potent IC_50_ values than the template compounds.

Furthermore, to test if our methods can be used for protein inhibitors other than type II kinases, E2020 series, the AChE inhibitors, with IC_50_ values varying from 0.9 to 4,400 nM, were selected for demonstrating the optimization of acetylcholine esterase inhibition activity [34]. We used an indanone-benzylpiperidine based compound with an IC_50_ value of 4,400 nM as the initial compound. As a result, compounds with better AchE inhibition activity were assembled using our methods.

Overall, this study demonstrates a novel template-based *de novo* design protocol to aid in the optimization of potent type II kinase inhibitors that preserved the original binding interactions of the template compound to the target and is capable of working with other protein targets.
